# Case of Epilepsy, Cured by Ligation of Both Carotids

**Published:** 1846-07

**Authors:** T. H. Hamilton


*Case of Epilepsy, cured by Ligation of both Carotids.* By T. H. Hamilton, Jr, M. D.

[We are indebted to Prof. Hamilton for the following interesting report.—*Ed. Buffalo Journal.*]

"The only successful case* on record in this country that we are aware of up to the present time, of a ligature on *both carotids* after a short interval of time, is that of Dr. Mott, in which both carotids were tied in an interval of 12 months. In another attempt of this kind by the same surgeon, in which there was an interval of only 15 minutes, the case ended fatally, (it produced coma) "we have now," Dr. T. farther remarks, "the pleasure of recording another triumph for American surgery, in the successful applica-cation of a ligature to both carotids after an interval of *four days* and a half." The operations were made in October last by Dr. Ellis of Michigan. Thus there are now on record five cases of the ligation of both carotids — Two by Dr. Mott, one by Dr. Mussey, one by Prof. Kuhl of Leipzig and one by Dr. Ellis. To which record we wish now to add one more successful operation by Dr. T. H. Hamilton, Jr. which operation, although it was made several years since, has never been published, and would probably have been entirely lost but for the following notes which at my request have been sent to me by his brother since his decease.

"Patient, a young man, aged 18, of a sanguine temperament; subject to epilepsy from childhood, the fits occurring at intervals of from two days to a month until he reached the age of twelve years. At this period, his disposition became very irritable, being frequently excited to violent anger; the fits also became more frequent until it was not uncommon for him to have a fit every day, and sometimes two or three in a day. During this time, he had been under a variety of medical treatment, both orthodox and heterodox, but without any improvement.

*First Operation.*—August, 1838—Present Dr. Christopher Kirsted and John Vedder, student of medicine, Dr. Hamilton having submitted the patient previously to a course of preparation, ligated the right common carotid.

This diminished, very materially, the frequency of the fits, and also their duration; indeed, they were not more than half as severe as before the operation.

*Second Operation.*—March, 1839—(six months from date of first operation,) present Drs. Martin Freligh, Christopher Kiersted and myself. My

---

* Quoted from Mott's Velpeau. A case has lately been reported by J. Mason Warren, M. D. See last No. of this Journal.

brother now ligated the left common carotid, which he found considerably enlarged in consequence of the circulation having been cut off in the right. The wound healed kindly, and since the last operation, nearly two years, the patient has not had a fit or a symptom threatening its occurrence.

VERONA, Oneida Co., Jan. 6, 1841.

Prof. F. H. HAMILTON:

SIR:—The preceding brief and imperfect sketch of the case of epilepsy, is, with pleasure, left at your disposal. I find, upon examination, that the intervals between the operations, were longer than I had stated in my thesis. I think the dates here given, are correct, as far as they go. If, however, I can find the notes which my brother left, I shall bring them to you, and leave them at your disposal.

Believe me sincerely and respectfully, your ob't, &c.

DEWITT C. HAMILTON.

I have not received any farther communication, but consider the case entirely reliable. F. H. H.

